# Supplementary material for: Combining magnetoencephalography with telemetric streaming of intracranial recordings and deep brain stimulation—A feasibility study
Source: Imaging Neurosci (Camb). 2023 Nov 7;1:imag-1-00029. doi: 10.1162/imag_a_00029 (PMC12007516; doi:10.1162/imag_a_00029)
Supplement: Supplementary Material [file imag_a_00029-supp.pdf]

Investigating the interaction between the OPM modulation signal and monopolar DBS stimulation

In the main manuscript we saw there were additional peaks in the spectrum when using monopolar stimulation with the OPM sensors compared the CTF-MEG SQUID system. We mentioned that many of the peaks could be determined by an interaction of the DBS device and the OPM modulation signal at 923 Hz. Here we show how these peaks can be determined by calculating the frequencies of nonlinear interaction between nearby spectral peaks to 923 Hz.

Figure S1 shows the spectrum of the OPM recording during monopolar stimulation 180 Hz either side of the modulation frequency (923 Hz; labelled peak 08). Eleven high amplitude peaks (and the modulation signal peak) have been identified by the vertical dashed lines. Note the threshold for selection is arbitrary but we selected any peaks above 15 fT/√Hz. Also note the QuSpin OPM sensors have 1st order low-pass filter from the vapour cell in the OPM starting at ~130 Hz and a second additional hardware-based 6th order low-pass filter at 500 Hz, so these peaks are considerably larger in amplitude than measured here.

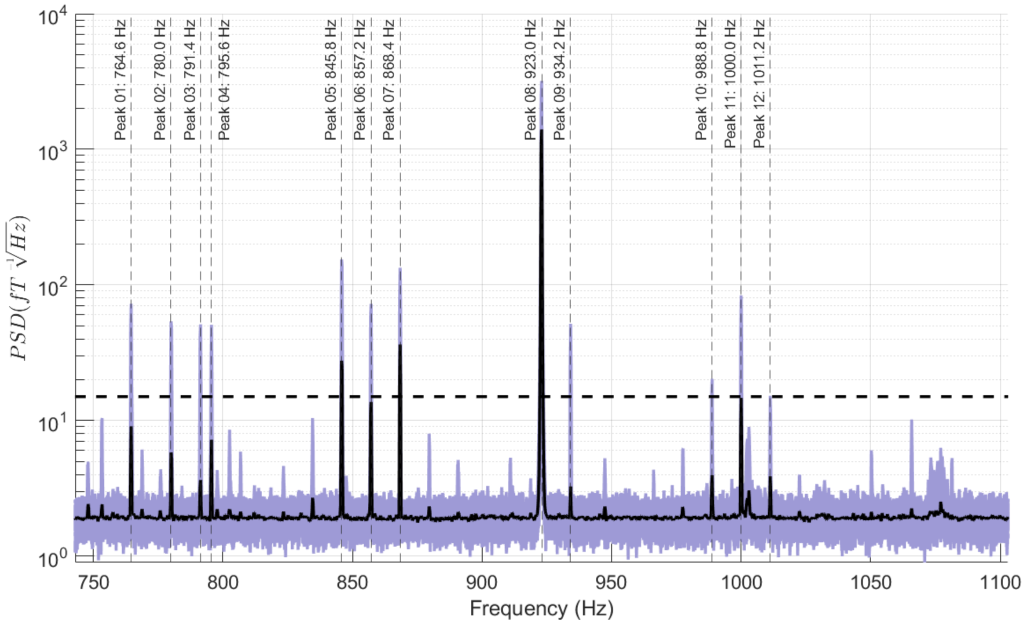

Figure S1: Spectrum of OPM sensor data 180 Hz either side of the modulation frequency (923 Hz) during monopolar stimulation. Here 11 peaks alongside the modulation frequency have been highlighted (vertical dashed lines). Purple lines represent the spectra of individual OPMs, the solid black line is the median spectral density (across all sensors) and the horizontal black dashed line represents 15 fT/√Hz, the threshold used to select peak frequencies for further analyses.

Our first assumption was that the difference in frequency between each of the 11 peaks and the modulation frequency would be represented in the low-frequency spectra. Figure S2 shows the spectrum of the OPM recordings between 0.1—180 Hz. There are multiple clear peaks which were not present when stimulation was not occurring (see main manuscript). Here we have overlaid the 11 lines representing where we would expect to find spectral peaks, as determined by the peaks near the modulation frequency (vertical dashed lines). We observe that these lines coincide with large peaks in the spectrum supporting that the idea the DBS is interacting nonlinearly with the modulation signal of the OPMs. However, this does not explain all the peaks, so we also looked at the

difference in frequency between each of the 11 high-frequency peaks to see if these predicted any other features in the spectrum. In Figure S3 we have overlaid predicted frequencies which coincided within 0.2 Hz of a peak in the spectrum (the spectral resolution of the fast Fourier transform used). Thirty nine predicted frequencies (out of a possible 55) were within 0.2 Hz of a peak, but for clarity we have overlaid 18 (for example two predicted frequencies were at 11.2 Hz and 11.4 Hz).

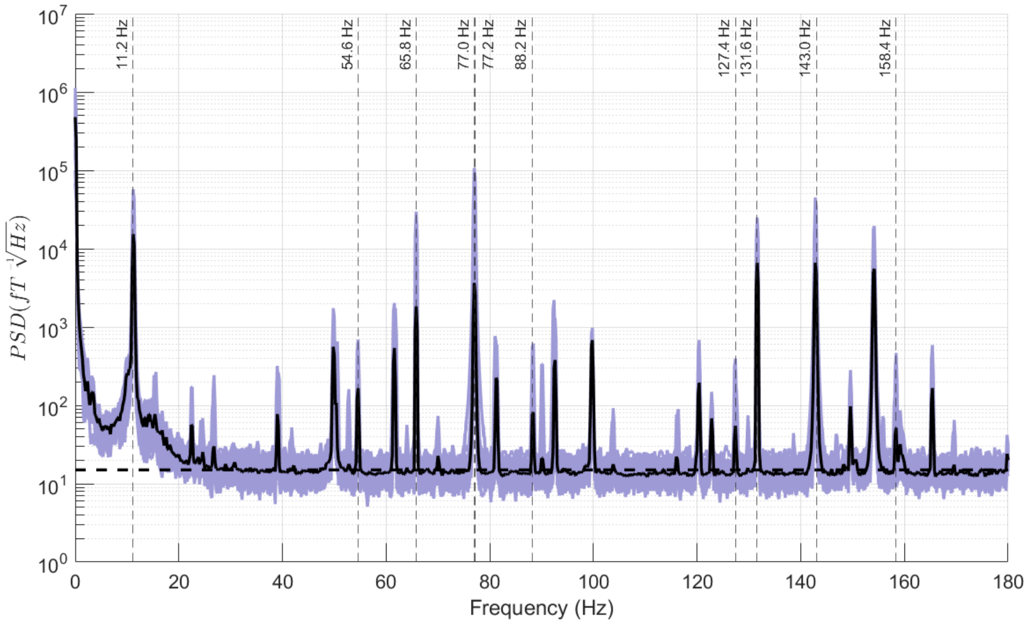

Figure S2: 0.1-180 Hz spectrum of the OPM sensors during monopolar stimulation. Overlaid as vertical dashed lines are the frequency differences between the high frequency peaks from Fig. S1 and the modulation frequency. The overlaid frequencies coincide with 11 peaks in the spectrum. Individual sensor spectra are in purple and the median spectra is a solid black line.

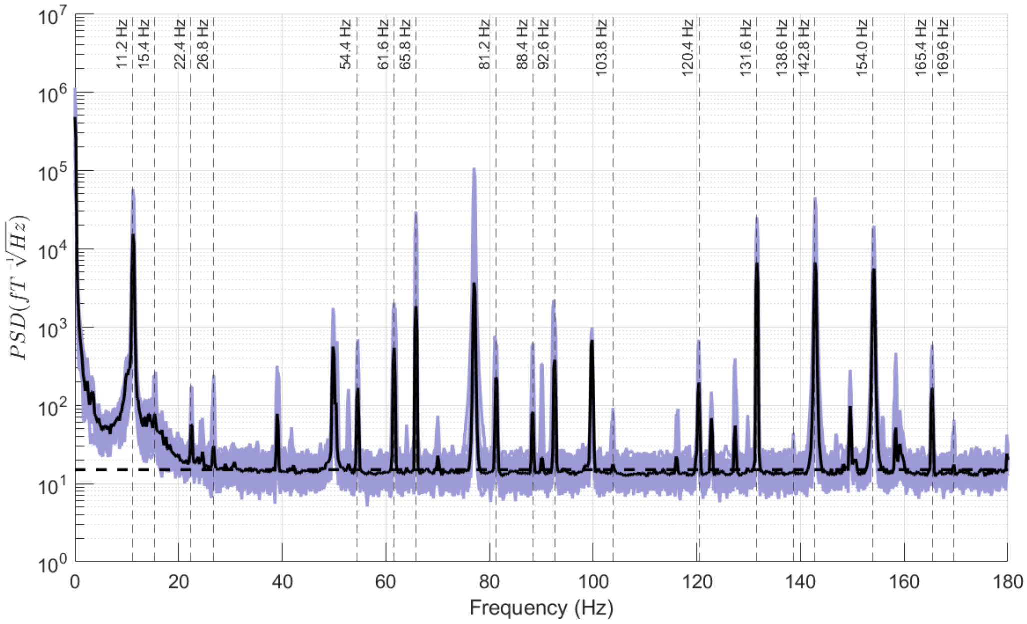

Figure S3: 0.1-180 Hz spectrum of the OPM sensors during monopolar stimulation. Overlaid as vertical dashed lines are the frequency differences between the high frequency peaks from Fig. S2, which coincide with large peaks in the low frequency spectrum. Individual sensor spectra are in purple and the median spectra is a solid black line.

Index	Percept mode	Movement	Streaming mode
1	Empty room	N/A	N/A
2	IPG off	No	N/A
3	IPG off	Yes	N/A
4	Bipolar stimulation	No	N/A
5	Bipolar stimulation	Yes	N/A
6	Monopolar stimulation	No	N/A
7	Monopolar stimulation	Yes	N/A
8	Indefinite streaming	No	SenSight
9	Indefinite streaming	Yes	SenSight
10*	Indefinite streaming	No	Legacy
11*	Indefinite streaming	Yes	Legacy
12	BrainSense OFF	No	SenSight
13	BrainSense OFF	Yes	SenSight
14*	BrainSense OFF	No	Legacy
15*	BrainSense OFF	Yes	Legacy
16	BrainSense ON	No	SenSight
17	BrainSense ON	Yes	SenSight
18*	BrainSense ON	No	Legacy
19*	BrainSense ON	Yes	Legacy

20	BrainSense ON 0	No	SenSight
21	BrainSense ON 0	Yes	SenSight
22*	BrainSense ON 0	No	Legacy
23*	BrainSense ON 0	Yes	Legacy
24	Open telemetry	No	SenSight
25*	Open telemetry	No	Legacy
26	BrainSense ON/OFF/ON	No	SenSight
27**	IPG off, communicator near the sensors	No	N/A

Supplementary Table S1. The experimental protocol using different sensing and stimulation modes of the Percept PC. *IPG off:* There is no stimulation and the communication session is closed, *Bipolar stimulation:* This is not a sensing mode, and data will not be streamed. The stimulation is done in bipolar stimulation configuration, *Monopolar stimulation:* same as Bipolar stim but in monopolar configuration. The testing is done in a case positive configuration (the IPG is the positive pole), *Indefinite streaming:* This option allows to stream data, recording LFPs on the electrodes in stim compatible pairs, which means contact pairs that are not immediately adjacent (such as 0-3, 1-3, and 0-2 on one hemisphere and 8-11, 9-11, 8-10 on the other). Stimulation is off during this measurement, *BrainSense OFF:* in this mode simultaneous stimulation and measurement of the LFPs using the contacts adjacent to the stimulating contact are enabled, using the BrainSense technology. The stimulation is done in monopolar stimulation configuration so that a geometrically symmetric configuration around the stimulating contact is possible. The stimulation artefact is, therefore, reduced due to the common mode rejection enabled by hardware design. Note that whilst stimulation in this mode is possible, one can also use it for LFP measurement with stimulation off, *BrainSense ON:* same as BrainSense OFF but this time the stimulation is turned on. In both the BrainSense and Indefinite streaming mode, the time domain data (i.e. LFP magnitude µV vs. time, sampled at 250Hz) is recorded as a JSON file on the clinical tablet. *BrainSense ON 0:* same as BrainSense ON but the stimulation amplitude is set to 0 mA. This mode is distinct from BrainSense OFF as there are switches that close for a duration equal to the pulse width and are open for the rest of the cycle. Open telemetry: the communication session is open but there is no stimulation and no recording. BrainSense ON/OFF/ON: stimulation was turned on and off several times in BrainSense mode to test for feasibility of its use as an alignment marker.

* These conditions were not tested on the MEGIN system.

** This condition was only tested on the CTF system
